# The outcomes of an audiological management programme for neonates with hyperbilirubinaemia

**DOI:** 10.4102/sajcd.v71i1.1014

**Published:** 2024-10-09

**Authors:** Moleboge M. Matshete, Samantha Govender, Sam T. Ntuli

**Affiliations:** 1 Department Speech-Language Pathology and Audiology, School of Health Care Sciences, Sefako Makgatho Health Sciences University, Pretoria, South Africa; 2 Department Statistical Science, School of Science and Technology, Sefako Makgatho Health Sciences University, Pretoria, South Africa

**Keywords:** hyperbilirubinaemia, hearing loss, auditory brainstem response, otoacoustic emissions, low birth weight, neonates, serum bilirubin level

## Abstract

**Background:**

Hyperbilirubinaemia is a contributing condition to the prevalence of neonatal hearing loss. Because of its pathophysiology, the use of Otoacoustic Emissions (OAE) and Automated Auditory Brainstem Response (AABR) testing is essential in diagnosing hearing loss. Two-tier screening models are typically used in developing world contexts; however, a combined approach to testing (using both tests) should be used for early detection. Blood serum levels should also be monitored to determine how they impact audiological test results.

**Objectives:**

To determine the outcomes of using a combined testing approach of both OAE and Auditory Brainstem Response (ABR) for both screening and diagnostic testing of neonates with hyperbilirubinaemia and studying the relationship between the test results and the serum bilirubin levels.

**Method:**

A cross-sectional, comparative design was utilised. Forty neonates were tested (80 ears). Neonates underwent hearing screening and diagnostic testing (ABR and/or AABR and DPOAE tests). The study was conducted at a hospital in South Africa.

**Results:**

One-third (32.5%) of the neonates had comorbidities. Screening results indicated that the AABR test could identify more cases of abnormalities than DPOAEs (*p* = 0.001). Participants with a serum level greater than 10 mg/dL presented with abnormal diagnostic ABR test results while passing the DPOAE test (*p* < 0.001).

**Conclusion:**

Combined use of ABR and DPOAE testing yielded a greater identification of auditory pathology than using either test alone. Serum bilirubin levels can be used as an indicator for combination testing.

**Contribution:**

Combined use of ABR and DPOAE testing leads to greater identification of auditory pathology.

## Introduction

The World Health Organization (WHO) estimates that 5% of the 34 million people who present with disabling hearing loss are children (WHO, 2022). There are various conditions that can cause hearing loss in children. In addition, there are many risk factors for hearing loss. One of the most predominant clinical conditions contributing to the global prevalence of neonatal hearing loss is hyperbilirubinaemia (Haile et al., 2021).

Hyperbilirubinaemia refers to a condition resulting in a build-up of bilirubin in the blood (Rafique et al., 2022). This results in yellow discolouration of the eyes and skin referred to as jaundice (Bradley, 2017). Hyperbilirubinaemia presents with an increased serum bilirubin levels often requiring exchange blood transfusion (Rafique et al., 2022). Bilirubin levels in neonates are evaluated by testing the levels of total serum bilirubin (TSB), bilirubin/albumin (B/A) ratio and unbound bilirubin in the blood (Wang et al., 2020). Hyperbilirubinaemia is treated with phototherapy and exchange transfusion. Research findings have indicated that the pathophysiology of hyperbilirubinaemia, and its associated treatment as well as concomitant conditions can cause hearing loss in neonates (Magai et al., 2020). A slightly increased level of bilirubin is usually not harmful to neonates; however, high levels of serum bilirubin levels have a harmful impact on the auditory system (Culhaoglu et al., 2021).

Literature describes that the peripheral system, auditory nerve, auditory nuclei within the brainstem, the inferior colliculus and the superior olivary complex are more vulnerable to the impacts of increased bilirubin levels (Teixeirat, 2020). Damage to these structures may lead to sensorineural hearing loss by impacting the peripheral and/or central auditory pathways (Teixeirat, 2020). Hyperbilirubinaemia reaching its toxic levels can affect the peripheral auditory system, particularly the cochlear or eighth nerve, the brainstem and/or the central nervous system, resulting in hearing loss and encephalopathy (Nam et al., 2019). Concomitant conditions alongside hyperbilirubinaemia, such as prematurity and low birth weight, can exacerbate hearing loss in this population (Appelbaum et al., 2018). In addition, the bilirubin levels in the blood can be a useful indicator for the need for screening and/or diagnostic testing to ensure the early identification of hearing loss. The most prevalent comorbidities are low birth weight, anaemia and respiratory distress disorder; these comorbidities affect the auditory neural maturation causing auditory pathology (Sundagumaran & Seethapathy, 2019). Because of the above-mentioned adverse effects of hyperbilirubinaemia on the auditory system, neonates presenting with this risk factor must have a comprehensive evaluation of their hearing.

Neonatal hearing screening protocols have been developed by various professional bodies including the Health Profession Council of South Africa (HPCSA), the Joint Commission of Infant Hearing (JCIH), WHO, American Association of Speech and Audiology (ASHA) and South African Speech-Language Hearing Association (SASLHA). Ideally, all babies should have their hearing screened at birth; however, because of resource constraints and poor awareness of the risks associated with unidentified hearing loss, many babies go undetected of hearing loss. The JCIH therefore recommends that in under-resourced countries, as a minimum, risk-based hearing screening should be conducted. These screening protocols outline that Otoacoustic Emissions (OAEs) are recommended for the testing of well babies, while Automated Auditory Brainstem Response (AABR) assessment is recommended for those babies who are at risk for hearing loss (SASLHA, 2011). Therefore, a two-tiered model for screening is recommended, in other words the use of the Distortion Product Otoacoustic Emissions (DPOAE) first, followed by the Auditory Brainstem Response (ABR) test if required. However, using the two-tier approach in patients with hyperbilirubinaemia may result in missing a hearing loss because of the pathophysiology of the disease (Vijayakumar, 2017).

A study was conducted by Shankar and Manjunath (2014) on 105 neonates diagnosed with hyperbilirubinaemia who underwent ABR and OAE testing. Of the 105 neonates, 99 passed the OAE, and six failed. With ABR, 96 neonates passed, and nine failed. This implied that three neonates would have been undiagnosed if ABR was not conducted. A similar study by Xu et al. (2011), including a larger population, also highlighted the importance of simultaneously utilising both TEOAE and AABR tests for hearing screening of neonates with risk factors. The study indicated that AABR had a significantly lower referral rate of 16.7% (24/144 neonates) than TEOAE (37.9%; 55/145 neonates). The literature also indicated that the blood serum levels can be an important decision-making factor on when to use the AABR alongside OAEs. However, there is a dearth of studies describing the correlation of blood serum levels with AABR testing. In a more recent study, Ezzeldin et al. (2021) conducted a study to establish the relationship between hyperbilirubinaemia at birth as a risk factor for neonatal hearing loss. In the study, 60 neonates were divided into two groups (group A and group B). Group A comprised neonates with hyperbilirubinaemia admitted to the NICU and receiving phototherapy or an exchange transfusion; group B included neonates without hyperbilirubinaemia. The results indicated that 22% of the neonates had failed the AABR and OAE. The results showed that using the OAE test alone might give a false impression of auditory pathology because the OAE test only measures the response of the outer hair cell and cochlear function. It does not account for damage to the neural pathways. Accordingly, it was recommended that OAE testing should not be used alone as a screening test; it should be used together with an AABR test in neonates with hyperbilirubinaemia. Ezzeldin et al. (2021) conducted a study to establish the relationship that exists between hyperbilirubinaemia at birth as a risk factor for neonatal hearing loss. The study was conducted on 60 neonates in which subjects were divided into two groups (group A and group B). Group A comprised neonates with hyperbilirubinaemia admitted to the NICU and were receiving phototherapy or exchange transfusion; the other group (group B) comprised neonates without hyperbilirubinaemia. The results indicated that 22% of neonates failed the AABR and DPOAE. The results indicated that using the DPOAE test alone may give a false impression of hearing loss because the DPOAE test measures the response of the outer hair cell and cochlear function only without accounting for the damage to the neural pathways. Therefore, it was recommended that DPOAE testing should not be used as a screening test alone but rather be used together with an AABR test in neonates with hyperbilirubinaemia. Thus, it is recommended to utilise both OAE and ABR in the screening and diagnostic testing when conducting audiological monitoring programmes on neonates with hyperbilirubinaemia.

The review of literature indicates that a combined use of DPOAE and ABR testing is not consistently used to manage patients with hyperbilirubinaemia despite the evidence suggesting that the disease can impact both the peripheral and neural pathways. In addition, the relationship between bilirubin levels and audiological test results has not been documented in the literature. In a resource-constrained context such as South Africa such recommendations of utilising both tests in this at-risk population must be substantiated with research-based evidence. Thus, the aim of the study was to evaluate the outcomes of an audiological management programme using a combined approach to testing (ABR and DPOAEs) in the identification and diagnosis of hearing loss in neonates with hyperbilirubinaemia. In addition, serum bilirubin levels and concomitant conditions were correlated to the audiological results to determine the relationship between the variables with the aim of using this information to guide decision-making into the use of combination testing.

## Research methods and design

### Study design

The study employed a quantitative approach using a prospective cross-sectional, within-subject comparative design where the screening and diagnostic AABR and DPOAE results were correlated for each participant. A total of 40 neonates were entered into the study based on parental consent, availability and overall wellness of the baby. From the 40 neonates, each ear was assessed resulting in a total of 80 samples/ears. The study was conducted at a tertiary academic hospital in South Africa. The inclusion criteria for this population included neonates (born less than 28 days)(Cnattingius et al., 2020), neonates with hyperbilirubinaemia and neonates born prematurely or full term. All risk factors in addition to hyperbilirubinaemia were recorded.

### Data collection

Participants underwent a hearing screening using AABR and DPOAEs, followed by diagnostic ABR and DPOAE testing. Testing took place in a counterbalanced manner in that screening commenced with a DPOAE test first then an ABR test and their diagnostic testing commenced with an ABR test and then a DPOAE test. This process continued for all participants in a counterbalanced manner. In addition, the serum bilirubin levels for all participants were measured and recorded to determine whether a relationship existed between high levels of bilirubin and diagnostic test results.

### Screening phase

Participants first underwent an otoscope examination. A healthy outer ear and external auditory canal and the presence of a light reflex from the tympanic membrane were landmarks for normal otoscope findings. With DPOAE testing, a probe was inserted into the ear canal and ABR testing skin cleaning and placement of electrodes on the forehead to conduct test. The test interpretation was a Pass/Refer criteria (Hall & Swanepoel, 2010). A pass result was recorded if a participant passed three or more frequencies. If a participant failed at two or more frequencies, then a refer result was recorded (Blankenship et al., 2018).

### Diagnostic phase

All participants underwent diagnostic testing. With regard to the interpretation of the diagnostic DPOAE results, the guidelines outlined in Hall and Swanepoel (2010) were used (Table 1).

**TABLE 1:** Interpretation of DPOAE results.

Variable	Category
Present OAEs within normal range	Most to all DP points fall within the normative region.
	Meaning: no outer hair cell damage is measured.
Present with reduced amplitude	Present but amplitude is below the normal limits (usually below 0 dBSPL)
	Meaning: outer hair cell damage occurs probably in the early phases.
Absent or not present	No evidence of OAE activity
	Meaning: definite outer hair cell damage occurs.

*Source:* Hall, J.W., & Swanepoel, D.W. (2010). *Objective assessment of hearing*. Plural publishing

OAE, Otoacoustic Emissions; DP, Distortion Product; DPOAE, Distortion Product Otoacoustic Emissions.

Regarding the ABR results, paediatric normative data as outlined by Hall and Swanepoel (2010) for both neurological and audiological tests were utilised (Table 2).

**TABLE 2:** ABR normative data for age group 3 months to 6 months.

Variable	Category	Mean	s.d.
Neurological norms	Latency of wave I at 80 dBnHL	1.59	0.171
	Latency of wave III at 80 dBnHL	3.73	0.214
	Latency of wave V at 80 dBnHL	6.523	0.321
	Interpeak Latency I–III	2.523	0.215
	Interpeak Latency III–V	2.218	0.215
	Interpeak Latency I–V	4.653	0.287
Audiological norms	Latency wave of wave V at 30 dBs	8.717	0.526

*Source:* Hall, J.W., & Swanepoel, D.W. (2010). *Objective assessment of hearing*. Plural publishing

ABR, Auditory Brainstem Response; s.d., standard deviation.

### Data analysis

Data were entered into Microsoft Office Excel© and later analysed using STATA version 11 (StataCorp, 2009). Data were analysed using both descriptive and inferential statistics. Descriptive analyses are presented using frequencies and percentages to determine the identification rate of possible hearing loss as well as the diagnosis of hearing loss. Blood results were analysed using two by two contingency tables. Concomitant conditions such as prematurity, birth defects, etc. were entered into cross-tabulation tables to determine whether the increase in concomitant conditions results in a pass or fail hearing test result. The Fisher’s exact test was used to study the relationship. The *p*-value was taken (*p* < 0.001) as statistically significant.

### Ethical consideration

Ethical clearance to conduct this study was obtained from the Sefako Makgatho University Research Ethics Committee (SMUREC) (reference no.: SMUREC/H/322/2021).

## Results

More than half (55.0%, *n* = 22) of the neonates were males and 45.0% (*n* = 18) were females. Of the neonates, 80.0% (*n* = 32) had low birth weight, and 47.5% (*n* = 19) were delivered by caesarean section. A total of 40 newborns were recruited for the study, of which 77.5% (*n* = 31) were premature neonates (Table 3). All the participating neonates were black African and most (67.5%, *n* = 27) of the neonates had blood serum > 10 mg/dL. One-third (32.5%) of the neonates had comorbidities. The demographic information of the study sample is displayed in Table 3.

**TABLE 3:** Demographic profile of participants (*N* = 40).

Variable	*n*	%
Gestational age < 37 weeks	31	77.5
Male gender	22	55.0
Birth weight < 2500 g	32	80.0
Delivery by CS	19	47.5
Blood serum levels < 10 mg/dL	13	32.5
Comorbidities	13	32.5

CS, Caesarian section.

### Objective 1: Screening and diagnostic outcomes

A total of 40 participants’ ears (*n* = 80 ears) were assessed using DPOAE and ABR testing. For those participants who were rescreened at one or more frequencies because of environmental noise or patient movement during testing, only the rescreen results were used for analysis. An otoscopic examination was conducted on all participants before the screening and diagnostic DPOAE and ABR testing. The results of the otoscopic examination indicated 100% (*n* = 80) normal outer and middle ears with the absence of any pathologies. Table 4 displays the frequency count of the pass, refer, normal and abnormal results obtained for the DPOAE and AABR screening results as well as the diagnostic OAE and ABR test results, respectively. For screening DPOAE test, more than half (56%) (*n* = 45) of the neonates obtained a normal result, and 44% (*n* = 35) obtained an abnormal result. This implies that there was a false-negative (FN) rate of 2 and a false-positive (FP) rate of 1. There was a true negative (TN) of 45 and a true positive (TP) of 35. All ears that failed the screening were given a rest period and re-screened to ensure the accuracy of the results. The results remained the same after the rest period.

**TABLE 4:** Pass, refer, normal and abnormal screening and diagnostic OAE and ABR.

Variable	Pass or normal†		Refer or abnormal‡	
	*n*	%	*n*	%
Screening DPOAE	64	80.00	16	20.00
Diagnostic DPOAE	66	82.50	14	17.55
Screening ABR	33	41.25	47	58.75
Diagnostic ABR	29	36.25	51	63.75

ABR, Auditory Brainstem Response; DPOAE, Distortion Product Otoacoustic Emissions; OAE, Otoacoustic Emissions.

† , normal OAE refers to no outer hair damage while normal ABR refers to normal auditory pathway.

‡ , abnormal OAE refers to outer hair damage while abnormal ABR refers to abnormal auditory pathway.

Regarding the AABR outcomes, 36.25% obtained an overall normal result, and 63.75% obtained an abnormal result, implying an FN of 2 and an FP of 2. The results also showed that the ABR testing was able to identify 46.25% more abnormal ears than the DPOAE test. This demonstrates that either test alone would have missed some ears with hearing loss and that the combined use of AABR and DPOAE yielded a better outcome and improved the disease accuracy. The AABR had a higher disease accuracy of 95.0% than DPOAE, which was 90.0%. In addition, disease accuracy measures were greater in combination than with either test alone (95.5%).

**FIGURE 1:**
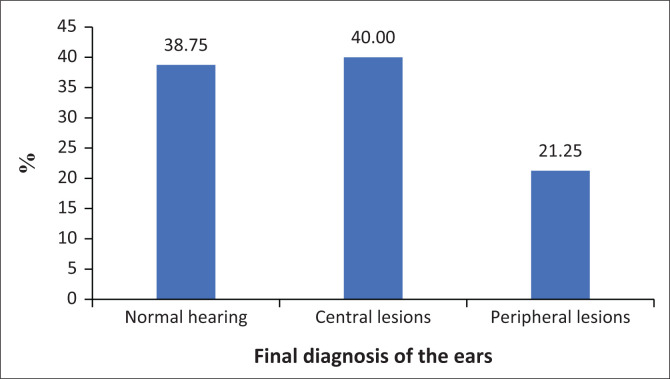
Diagnosis made after the diagnostic testing.

Table 5 displays the normal and abnormal results of the diagnostic DPOAE and ABR tests. The results indicate that ABR testing was able to identify more abnormal cases than the DPOAE test (DPOAE: *n* = 14, ABR: *n* = 37). Furthermore, in combination, the ABR and DPOAE tests could identify a higher percentage of pathology than by using either test in isolation (*n* = 51). Figure 1 illustrates the final diagnosis of the ears (*n* = 80). A total of 38.75% (*n* = 31) had normal hearing, 40% (*n* = 32) had central lesions and 21.25% (*n* = 17) presented with peripheral lesions. Thirty-three neonates presented with a conductive hearing loss (CHL). From the 32, 11 presented with severe CHL and 22.

**TABLE 5:** Comparison of abnormal rates of diagnostic DPOAE and ABR results.

Diagnostic DPOAE	Diagnostic ABR		Total
	Abnormal‡	Normal†	
Abnormal‡	14	0	14
Normal†	37	29	66

**Total**	**51**	**29**	**80**

DPOAE, Distortion Product Otoacoustic Emissions; ABR, Auditory Brainstem Response.

† , normal ABR refers to normal auditory pathway.

‡ , abnormal ABR refers to abnormal auditory pathway.

presented with mild to moderate CHL. Sixteen neonates presented with a mild-to-moderate sensory neural hearing loss.

### Objective 2: Relationship between audiological diagnostic test results in neonates with hyperbilirubinaemia and the level of bilirubin concentration in the blood

A correlation of DPOAE and ABR diagnostic test results in relation to the serum bilirubin levels was made to determine if there was a relationship between abnormal results and elevated serum bilirubin levels. As shown in Table 6, in the ears of patients with a blood serum level of 10 mg/dL or more, there was an abnormal audiological result of 68% (*n* = 54). The results further indicated that in patients with an abnormal ABR result, the serum levels were usually greater than 10 mg/dL, and the results also revealed that more participants with a serum level greater than 10 mg/dL obtained an abnormal result for the ABR test and obtained a normal result for the DPOAE test, which was also statistically significant (*p* < 0.001). This could suggest that an ABR test is at least required for the screening and diagnostic testing of patients with hyperbilirubinaemia with a serum bilirubin level of > 10 mg/dL if it cannot be utilised uniformly for all patients with hyperbilirubinaemia. Table 6 shows the comparison of diagnostic test results in relation to serum bilirubin levels.

**TABLE 6:** Comparison of diagnostic test results in relation to bilirubin level.

Variable	Diagnostic OAE				Diagnostic ABR			
	Blood serum ≥ 10 mg/dL		Blood serum < 10 mg/dL		Blood serum ≥ 10 mg/dL		Blood serum < 10 mg/dL	
	*n*	%	*n*	%	*n*	%	*n*	%
Abnormal	11	20.4	3	20.4	48	88.9	3	11.5
Normal	43	79.6	23	79.6	6	11.1	23	88.5

**Total**	**54**	**100.0**	**26**	**100.0**	**54**	**100.0**	**26**	**100.0**

Note: Normal OAE refers to no outer hair damage while normal ABR refers to normal auditory pathway; abnormal OAE refers to outer hair damage while abnormal ABR refers to abnormal auditory pathway.

OAE, Otoacoustic Emissions; ABR, Auditory Brainstem Response.

The majority of the participants in the study were found to have central (40.0%) lesions followed by peripheral lesions (21.3%) and only 38.8% had normal findings.

### Summary of the results

The screening results indicated that the ABR test could identify more cases of abnormalities than the OAE test, which was found to be statistically significant (*p* = 0.001), and that the combined use of ABR and DPOAE testing yielded a greater identification of auditory pathology in the study sample than using either test alone. As shown in Table 6, in the ears of patients with a blood serum level of 10 mg/dL or more, there was an abnormal audiological result of 68% (*n* = 54). The results further indicated that in patients with an abnormal ABR result, the serum levels were usually greater than 10 mg/dL and the results also revealed that more participants with a serum level greater than 10 mg/dL obtained an abnormal result for the ABR test and obtained a normal result for the DPOAE test, which was also statistically significant (*p* < 0.001). The majority of participants presented with neural lesions/pathologies.

## Discussion

In this study, it was found that both tests in combination were more useful than using either one as their combination increased the disease accuracy. The use of both tests yielded a higher percentage of identification of auditory pathology. This finding aligns with the literature in terms of pathophysiology. The neural toxicity in hyperbilirubinaemia can affect the cochlear hair cells and neurons of the basal ganglia and the central auditory pathway. Without utilising the DPOAE means missing a possible damage to the outer hair cells (Martínez-Cruz et al., 2014) and limited utilisation of the ABR test could miss possible damage to the central auditory pathway. The findings of the study are similar to that conducted by Halder et al., (2022) where the AABR test yielded a higher sensitivity rate than OAE testing. The findings suggest that the combined use of both tests is essential to reduce FNs and false positives. Furthermore, the combined use of both OAE and AABR is crucial in the identification of the Auditory Neuropathy Spectrum Disorder (ANSD). Early identification of ANSD is crucial because bilirubin-associated ANSD can be transient, and some neonates hearing can be progressively returned to normal by lowering bilirubin levels and administering nutritional neuro-drugs. The improvement in ANSD is observed from 12 months – 18 months of age (Chen et al., 2023). The combined use of measures therefore improves the early detection and identification of ANSD in this population.

The study’s findings further indicated that in patients with a bilirubin level of > 10 mg/dL, the ABR test was more sensitive in detecting auditory pathology. This could be because the literature indicates that serum bilirubin levels > 10 mg/dL have a greater impact on the neural pathways (Culhaoglu et al., 2021). The findings of this study could serve as a recommendation for audiologists in terms of when to use both ABR and DPOAE testing. Resource constraints are an issue; therefore it is recommended that audiologist should consider this cut-off level of > 10 mg/dL as when to use combined testing of both OAE and ABR when testing neonates with hyperbilirubinaemia. However, a study conducted by Ezzeldin et al. (2021) suggested a bilirubin level cut-off level of 21 mg/dL causes the value correlated with more abnormal ABR results. Another study found that a cut-off level of > 11 mg/dL is essential as neonates with > 10 mg/dL had presented with abnormal ABR (Akinpelu et al., 2013). Because of the challenges faced in South Africa with regard to limited equipment, few audiologists and noisy environments, it will be difficult to conduct a combined approach all the time. Therefore, audiologist should have access to the blood results when testing neonates with hyperbilirubinaemia, which could guide their decision-making test selection.

### Recommendations

The result of this study suggests the combined use of both ABR and OAE is useful while testing neonates with hyperbilirubinaemia, particularly for those with a bilirubin level of > 10 mg/dL. Audiological monitoring programmes should consider combination testing to improve outcomes. Further studies on the serum bilirubin level are required to standardise the blood serum level.

## Conclusion

Routine audiological management is required for patients with hyperbilirubinaemia. The combined use of ABR and DPOAE tests yields a higher identification of auditory pathology than either test in isolation. ABR did appear to be more sensitive in detecting auditory neuropathy. There was a correlation between bilirubin levels and the ABR response. A cut-off point of 10 mg/dL TSB levels equal to or above this value correlated with an abnormal ABR test and higher levels of TSB were associated with a higher risk of hearing impairment. This information can be assimilated into the decision-making when designing audiological monitoring programmes for patients with hyperbilirubinaemia.
